# Marginal Ulcers after Roux-en-Y Gastric Bypass: Etiology, Diagnosis, and Management

**DOI:** 10.3390/jcm12134336

**Published:** 2023-06-28

**Authors:** Marita Salame, Noura Jawhar, Amanda Belluzzi, Mohammad Al-Kordi, Andrew C. Storm, Barham K. Abu Dayyeh, Omar M. Ghanem

**Affiliations:** 1 Department of Surgery, Mayo Clinic, Rochester, MN 55905, USA; 2 Division of Pediatric Surgery, UPMC Children’s Hospital of Pittsburgh, Pittsburgh, PA 15224, USA; 3 Department of Gastroenterology and Hepatology, Mayo Clinic, Rochester, MN 55905, USA

**Keywords:** marginal ulcer, Roux-en-Y gastric bypass, bariatric surgery

## Abstract

Marginal ulcer (MU) is a potential complication following Roux-en-Y gastric bypass (RYGB), with a mean prevalence of 4.6%. Early identification and prompt intervention are crucial to mitigating further complications. The pathophysiology of MU is complex and involves multiple factors, including smoking, *Helicobacter pylori* infection, non-steroidal anti-inflammatory drug (NSAID) use, and larger pouch size. Patients with MU may experience acute or chronic abdominal pain. Rarely, they may present with a complication from the ulceration, such as bleeding, perforation, or strictures. Following diagnosis by endoscopy, management of MU typically involves modification of risk factors and medical therapy focused on proton pump inhibitors. In case of complicated ulcers, surgical intervention is often required for the repair of the perforation or resection of the stricture. For recurrent or recalcitrant ulcers, endoscopic coverage of the ulcer bed, resection of the anastomosis, and abdominal or thoracoscopic truncal vagotomy may be considered. This review aims at providing an overview of the etiology, diagnosis, and management of MU after RYGB.

## 1. Introduction

Obesity is a chronic and complex disease that has been rapidly increasing in prevalence over the years. The World Obesity Federation’s 2023 Atlas predicts that more than half of the global population will be affected by obesity within the next 12 years [1]. While conservative treatments and lifestyle changes can help manage obesity, metabolic and bariatric surgery (MBS) is currently considered the most effective long-term treatment [2,3]. Roux-en-Y gastric bypass (RYGB) is one of the most commonly performed MBS procedures, showing sustained weight loss outcomes and comorbidity resolution [4,5,6]. While rare, RYGB can entail risks, including the development of long-term complications such as anastomotic strictures, internal herniation, and marginal ulcers (MU) [7,8].

MU is a potential complication following RYGB, which typically occurs at or near the gastrojejunal anastomosis, with a reported mean incidence rate of 4.6% [8,9,10]. While the pathophysiology of MU remains unclear, there are several potential risk factors that have been identified, including smoking, diabetes, use of nonsteroidal anti-inflammatory drugs (NSAID), *Helicobacter pylori* infection, and alcohol consumption, among others [10,11,12,13,14,15,16,17]. Symptoms of MU can vary, with some patients being asymptomatic while others present with nausea/vomiting, abdominal pain, or gastrointestinal (GI) bleeding, potentially explaining the variability in the reported incidence rates [8,18]. In severe cases, MU may perforate and require urgent treatment [8,19]. The timing and severity of presentation often dictate the appropriate course of treatment [10]. Therefore, early detection and prompt management, whether medical or surgical, are crucial to ensuring a favorable outcome.

In this review, we provide an overview of MU after RYGB and potential management strategies based on available data. We performed an extensive electronic database search using PubMed, Scopus, MEDLINE, and Google Scholar for studies on the topic from inception to 1 March 2023 in English. We prioritized more recent, higher impact studies with larger cohorts when possible.

## 2. Epidemiology

The mean incidence of MU after Roux-en-Y gastric bypass (RYGB) is reported to be 4.6%, according to a systematic review of 41 studies. In these studies, the time between surgery and the presentation of MU varied from 1 month to 6 years. However, it is worth noting that, in certain series, the incidence can be as low as less than 1% [18,20]. The reported incidence is extremely variable, and these differences in incidence rates are likely attributed to the absence or presence of symptomatology, the duration of follow-up after RYGB, and the duration until MU development post-surgery. Typically, MU develops 6–12 months after the surgery. In a recent systematic review of 16 different observational studies, the incidence rate of MU was reported to range between 2.3% and 18.6%, with diagnosis starting as early as 14–16 days and as late as 20 years [21]. El Hayek et al. concluded that early MU, diagnosed within the first 3 months of the postoperative period, is likely a component of the normal process of anastomotic healing. They also found that approximately 50% of their diagnosed MU cases were classified as late MU, having been detected more than 12 months following RYGB [22].

## 3. Physiopathology and Predictors

The pathophysiology of MU is multifactorial and remains not fully understood. While the exact cause is unclear, some possible mechanisms have been suggested. One of the primary culprits is the presence of highly acidic gastric secretions, which has been linked to the formation of gastrojejunostomy (GJ) ulcers in Billroth procedures that involve parietal-cell-rich portions of the stomach [23]. Following RYGB, antral stimulation and gastrin release are decreased as food bypasses the antrum. Nevertheless, the gastric mucosa remains capable of responding to vagal and hormonal stimuli, thereby maintaining an acidic environment [24]. The jejunal mucosa, which lacks protective buffering mechanisms, is vulnerable to the effects of gastric secretions [25]. Local activation of pepsin in the jejunal mucosa is believed to be triggered by the high acidity of gastric secretions, leading to the development of MU [26]. In addition, the formation of gastrogastric fistula after RYGB has been found to lower the pH in the gastric pouch. This occurs as acid from the gastric remnant flows through the fistula and into the pouch, contributing to the development of MU [17,27]. Several other potential risk factors, such as comorbidity-related (diabetes, *H. pylori*) and lifestyle-related factors (smoking, NSAID use, alcohol consumption), have been suggested to impair tissue perfusion and cause local ischemia and chronic inflammation. Furthermore, the use of serotonin reuptake inhibitors (SSRIs) has been associated with an increased risk of ulcer bleeding. This may be attributed to the anticoagulant properties and potential ulcerogenic effects of SSRIs on the intestinal mucosa, which can affect platelet function and increase the likelihood of bleeding [28].

### 3.1. Anatomic and Surgery-Related Factors

MU formation is influenced by the size of the gastric pouch, as larger pouches contain a greater parietal cell mass that produces acid, resulting in increased acidity levels and a higher likelihood of developing ulcers [29,30]. It has been suggested that inflammation-inducing conditions and prolonged irritation from foreign materials, such as the use of non-absorbable suture material, staples leading to staple line dehiscence, and tension on the anastomosis, can increase the risk of MU. Conversely, the use of absorbable sutures and the creation of a smaller gastric pouch (less than 50 mL) can decrease its risk [31,32]. Studies have compared the occurrence of MU between non-absorbable and absorbable sutures, revealing a substantial decline in incidence from 2.6% to 1.3%, respectively [33]. Ayuso et al. found that the incidence of MU is positively correlated with the mean gastric pouch size, with a doubling of the risk for every 5 cm^3^ increase in size [34].

Several studies have also reported that circular stapled anastomosis (CSA) resulted in higher rates of MU development when compared to linear stapled anastomosis (LSA) and hand sewn anastomosis (HSA) [35,36,37,38]. Lois et al. found a significant difference in the occurrence rate of MU in CSA (5.5%) when compared to HSA (0.7%), whereas Major et al. also found a higher MU occurrence rate with CSA (10.3%) when compared to LSA (2.1%) [37,38]. Regardless of improvements in surgical techniques, continued development of novel surgical methods for RYGB, and the introduction of prophylactic regimens, MU continues to prevail, suggesting the role of other patient-related factors.

### 3.2. Helicobacter pylori

*Helicobacter pylori* is known to play a prominent part in the development of gastric and duodenal ulceration and gastric malignant lesions [39].The exact role of *H. pylori* in post-RYGB ulcer pathogenesis remains unclear. It is believed that the bacteria could potentially create a state of chronic inflammation accompanied by gastritis and metaplasia, ultimately resulting in the formation of MU [40]. While some studies report no association between *H. pylori* infection and the occurrence of MU [41,42], more solid data reveals a substantial correlation between the presence of the bacteria and MU [11,43,44]. This was further corroborated by Beran et al., who identified *H. pylori* infection as the most significant predictor for MU [11].

The identification of *H. Pylori* in patients who have undergone RYGB can be challenging, as urea breath tests may yield false negative results due to limited contact with the gastric remnant mucosa [45]. While serology has limited diagnostic value, monoclonal stool antigen tests have a sensitivity and specificity of over 90%, making them the most suitable non-invasive diagnostic tool [39,46]. Histological samples, however, remain the gold standard for accurate detection. In revisional cases, biopsies should also be taken to ensure accurate detection. Given the influence of *H. pylori* on MU formation, preoperative screening, and eradication of *H. pylori* before bariatric surgery may help minimize the incidence and development of subsequent *H. pylori*-associated ulcers.

### 3.3. Diabetes

Several studies have identified diabetes as a significant predictor of MU [8,10,11,17,47]. The increased risk of MU in diabetic patients may be due to various mechanisms, such as insulin resistance leading to the overexpression of proinflammatory mediators, vasoconstriction induced by circulating fatty acids, and activation of prothrombotic factors, all of which result in tissue perfusion impairment [28]. In their study, Süsstrunk et al. found that for each unit rise in HbA1c above 6.0%, there was a 23% increase in the risk of developing MU [8]. Therefore, optimizing glycemic control before and after surgery is crucial for reducing the postoperative risk of MU.

### 3.4. Smoking

The association between smoking and the increased risk of developing peptic ulcers among the general population has been well established for a long time [48]. Molecular studies have demonstrated that smoking can cause mucosal cell death, inhibit cell renewal, decrease blood flow in the GI mucosa, and interfere with the mucosal immune system [49]. Several studies have found a significant association between tobacco use and MU formation following RYGB [8,10,11,13,20]. Dittrich et al. found that smoking was a significant predictor for the development of MU, with a 4.6-fold higher risk [13]. Experts recommend that individuals who are considering bariatric surgery refrain from smoking for at least six weeks preoperatively [50].

### 3.5. Non-Steroidal Anti-Inflammatory Drugs

The use of NSAIDs impacts the GI mucosa primarily by inhibiting cyclooxygenase, reducing prostaglandins, and decreasing blood flow, which leads to a decrease in bicarbonate and mucus secretion [51]. While multiple studies have reported an increased risk of MU with NSAID use [14,20,44,52], others have found no association [17,22,28]. This variation might be due to differences in study designs, NSAID types and dosages, duration of use, and patient reporting. Several studies have demonstrated that short-term use (<30 days) and low doses of NSAIDs and aspirin may not increase the risk of MU, whereas higher doses and chronic use do [28,44,52]. Therefore, it is recommended to avoid using NSAIDs after surgery, as they may potentially contribute to the development of MU. However, low-dose aspirin may be warranted for patients with a medical indication for its use following RYGB. In these cases, we recommend combining low-dose aspirin therapy with proton pump inhibitors (PPIs).

### 3.6. Alcohol Use

Studies have presented contradictory evidence of the role of alcohol in MU. While it has been previously assessed as a risk factor for MU in patients post RYGB, the results have not been significant during statistical analysis despite its prevalence. A recent meta-analysis, including five recent studies by Beran et al., concluded that alcohol use was not linked to a higher risk of MU [11]. However, Wynn et al. discovered that consuming more than one alcoholic beverage per day and presenting with a perforated MU increased the risk of ICU admission following RYGB [53]. Moreover, Boelarge et al. found a strong correlation between alcohol use and significant MU findings during upper endoscopy after RYGB in symptomatic patients [20]. In fact, alcohol consumption can disrupt this equilibrium by causing dysbiosis, which undermines the role of the microbiome in maintaining the epithelial barrier [54]. Given the potential adverse effects of alcohol on the GI mucosa and its detrimental effects on the health of the patients who underwent bariatric surgery, it is highly advisable to abstain from alcohol consumption following RYGB surgery.

## 4. Diagnosis

MU is typically confirmed through upper endoscopy, which is the preferred and widely accepted gold standard diagnostic method [12,17,55]. On endoscopy, the ulcer usually appears as a well-defined area of tissue loss or erosion, with a smooth base and edges. The surrounding mucosa may appear inflamed, and visible blood vessels may be present. MUs can be single or multiple, and their size can vary from a few millimeters to several centimeters [8]. Biopsies may also be taken during endoscopy to confirm the diagnosis and rule out other potential causes of the symptoms [56]. Endoscopy can also reveal various findings, such as gastrojejunal strictures, gastrogastric fistulas, enlarged pouch, extruded surgical suture material or staples, and perforation of the pouch or anastomosis [55].

Several studies have identified the most frequent sites for MU formation on upper endoscopy. Bacoeur-Ouzillou et al. found that the most common location of MU was the gastrojejunal anastomosis site, with an occurrence rate of 71.4%, followed by the jejunal limb adjacent to the anastomosis, with a rate of occurrence of 12.5% [57]. Similarly, Azagury et al. reported that 50% of MU occurred at the gastrojejunal anastomosis, 40% at the proximal jejunal limb adjacent to the anastomosis, and 10% in the gastric pouch [17]. Overall, these studies consistently demonstrate that the gastrojejunal anastomosis site and the jejunal limb adjacent to the anastomosis are the most common sites for MU formation after RYGB.

While most studies support the use of endoscopy as the best diagnostic method for MU, a few studies also suggest that GI series may be used as an additional diagnostic tool. In cases where RYGB patients present with symptoms in the emergency or outpatient setting, a computerized tomography (CT) scan is the most commonly used imaging modality to identify the etiology of the presenting symptoms and exclude more sinister issues, such as perforation, hernia, intussusception, or bowel obstruction [19,21,58,59]. A retrospective cohort study conducted by Zulfiqar et al. reported that CT scan findings of focal bowel wall thickening, mucosal defect/outpouching, and fat stranding were the three most reliable imaging indicators of MU. Fat stranding was the most reliable feature and was found to have the highest prevalence among MU patients, and subsequent endoscopy confirmed the diagnosis in all included MU patients [58]. While a CT scan may not definitively diagnose MU, its findings should prompt further investigation with endoscopy, especially since it is commonly used as an initial imaging modality in symptomatic post-operative RYGB patients [59]. This approach could lead to an early diagnosis of MU, prompting aggressive therapy and preventing further complications, such as MU perforation.

## 5. Management

### 5.1. Medical Therapy

The management of MU initially entails the modification of the non-surgical risk factors, such as smoking, the use of nonsteroidal anti-inflammatory drugs, and alcohol consumption, and administering acid-reducing medications such as PPIs, H2 blockers, and sucralfate [60,61]. However, the appropriate regimen, dose, and duration of prevention are still controversial [62]. The length of initial treatment varies from 1 week [63] to 3 years [64]. Coblijn et al. found that acid suppression therapy for 6 months after surgery lowered MU rates from 7.3% to 1.2%, while Kang et al. demonstrated that a 90-day treatment was superior to only 30 days of treatment in preventing ulcers [65,66]. The healing rate of MU through lifestyle changes and medical therapy ranges from 68% to 100%, but relapse rates of up to 8% have been reported [21,67,68,69].

The prevailing approach to treating ulcers, except in emergency cases such as gastrojejunal perforation, involves a medical approach based on the use of PPIs, due to the hypothesis that acidity in the gastric pouch contributes to ulcer formation. A study by Dallal and Bailey employed a step-down treatment approach that involved a month of high-dose PPIs and sucralfate, which was gradually reduced on a monthly basis. They noted that sucralfate was more effective than PPI therapy, although this was only based on anecdotal evidence [70]. Interestingly, Azagury et al. discovered comparable healing rates of 67% and 68% when comparing PPI monotherapy and PPIs with sucralfate [17].

Recent data portrays the limited effectiveness of PPI capsules in RYGB patients due to the small size of the gastric pouch and the rapid transit time of the small bowel. A study compared the delivery of PPIs via an open capsule approach to intact capsule PPIs and found that the open capsule approach significantly reduced ulcer healing times. Those who received open-capsule PPIs had a median time of 3 months for ulcer healing, while those who received an intact capsule formulation had a median healing time of approximately 11 months [43]. Because of this, it is the authors’ practice to advise patients with RYGB-associated MU to open the PPI capsule before taking it by mouth. Lifestyle modifications and the combination of open-capsule PPI therapy with sucralfate form a robust primary therapy for MU.

### 5.2. Endoscopic Therapy

Endoscopic procedures, such as endoscopic suturing and stenting, have become popular as minimally invasive step-up therapies to consider prior to revisional surgery for managing non-perforated MUs that have not responded to lifestyle or medical therapy [71]. These procedures can be safely used in cases of non-healing MU, deep-penetrating ulcers at risk of perforation, and MU bleeding that does not respond to conventional endoscopic therapy, such as coagulation or endoscopic clips. The authors have also used endoscopic suturing and/or stent placement for treatment of selected hemodynamically stable patients with MU with microperforation.

Some reports suggest that oversewing using an endoscopic suturing system can be successful in healing ulcers [72,73]. Barulo et al. managed a series of 11 MUs endoscopically using endoscopic suturing and/or stent deployment, with no complications during or after the procedure [74]. The exact mechanism by which ulcer oversewing works to heal ulceration is unclear, but it may protect the lesion from exposure to irritating gastric pouch contents and increase the likelihood of healing [73]. Similarly, fully covered self-expandable metallic stents, including the more recently described lumen-apposing metal stent, may also be a viable option for treating MU, especially when the gastric outlet is too narrow for endoscopic oversewing [74].

### 5.3. Surgical Treatment

Revisional surgery should be considered in patients who have MU refractory to medical treatment, have recurring cases despite successful treatment, or have associated gastrogastric fistulas, which allow the retrograde reflux of acid through the fistula without the benefit of buffering from alkaline bicarbonate-rich pancreatic juice, as occurs naturally in the duodenum. Additionally, lifestyle modification, such as cessation of smoking, is essential, since it has a significant impact on preventing the occurrence of MU or managing refractory MU. Failure to address refractory MU promptly can result in serious complications, such as overt bleeding, perforation, intractability, or stricture, which can rapidly escalate into a surgical emergency and lead to peritonitis, sepsis, and potentially fatal outcomes [61].

The incidence of patients with ulcers who ultimately require surgical intervention to aid in ulcer resolution is reported to be between 3.9% and 33% [15,17,67,68]. However, a consensus on the optimal procedure for addressing recurrent MU has yet to be reached. A survey conducted by Steinemann et al. showed a lack of consensus among surgeons in managing MU after RYGB, with 56% choosing medical treatment, 41% opting for anastomosis revision, and 2% performing total gastrectomy and esophagojejunostomy for recurrent ulcers [61].

Various surgical approaches have been suggested, such as the resection of the GJ, including the ulcer and redo of the anastomosis alone [10,17,53,67], GJ revision with vagotomy (transthoracic or transabdominal) [69,75,76,77], subtotal or total gastrectomy [55,78], reversal of gastric bypass [79,80,81], and RYGB conversion to sleeve gastrectomy [64]. These procedures aim to correct different contributing factors, such as reducing acid production through vagotomy and correcting mucosal disruption, ischemia, and gastric pouch acidity by making the pouch smaller with GJ revision [10,17,55,69,75]. Studies suggest that one of the most effective approaches is a combination of GJ revision and/or abdominal or thoracoscopic truncal vagotomy [21,57]. When medical therapy for MU fails, most surgeons recommend removing the ulcerated GJ and restoring the GI integrity through hand-sewn or stapled reanastomosis to promote healing in revisional surgery for refractory and ischemic (when suspected in the first 30 days post-RYGB) MU [55,67,76,82]. Vagotomy may be necessary if acidity is suspected to be a contributing factor. In a recent study conducted by Chang et al., a revision using hand-sewn GJ along with a truncal vagotomy was performed on 11 patients, with 100% resolution and no reported recurrence of MU [69]. Sometimes, the only feasible option may be to remove the entire pouch and perform an esophagojejunostomy when the pouch is already small or a gastrogastric fistula is present. In a study by Chau et al., 10 patients underwent subtotal gastrectomy, and one underwent total gastrectomy with esophagojejunal anastomosis, all resulting in a 100% resolution rate with no recurrence [55].

If a patient is unable to quit smoking and experiences persistent MU or other significant complications (such as hypoglycemia) after RYGB, a bypass reversal may be required [79,80,81]. Although reversal to normal anatomy resolves MU-related issues, 30–40% of patients may still experience symptoms after the reversal, as seen in studies by Ma et al. and Moon et al. [79,83]. An alternative approach for patients who still want to benefit from a weight loss procedure is converting gastric bypass to sleeve gastrectomy after reversal. Carter et al. reported close to 60% overall morbidity and 25% Clavien–Dindo 3+ morbidity without information on symptom resolution [64]. Therefore, further research is needed to evaluate the safety and feasibility of this approach.

## 6. Complicated Ulcers

### 6.1. Perforation

For patients who present with perforation, the surgical approach focuses on addressing the perforation itself, followed by medical therapy, risk factor optimization (smoking cessation, discontinuation of NSAIDs, *H. Pylori* eradication, etc.), and endoscopic surveillance. Endoscopic surveillance is important, given the high rate of recurrence following surgical treatment [15]. The optimal surgical treatment for perforated MU remains controversial, as there is no consensus among experts. Several studies have evaluated different surgical management strategies, such as open and laparoscopic closure with an omental patch or anastomotic revision [19,84,85]. Wendling et al. showed that omental patch repair can be safely applied to treat perforated ulcers [84]. Nonetheless, if the ulcer was caused by an ischemic ulcer, a large gastric pouch, or an occult gastrogastric fistula, the root problem may still exist. To tackle these potential complications, a revision GJ can be performed. A recent study by Crawford et al. found that revision GJ is a safe and effective approach for perforation after RYGB, with a lower chance of ulcer recurrence and similar short-term morbidity compared to suturing with or without an omental patch [86]. Our preference is to manage the perforation with omental patching and then decide on anastomotic revision (if needed) in an elective setting. Endoscopic management may be a viable option for a contained perforation before resorting to surgery. Barola et al. have demonstrated its safety and technical feasibility while showing promising outcomes for future consideration [72].

### 6.2. Strictures

Strictures can develop at the gastrojejunostomy at the ulcer site after RYGB [33]. Symptoms such as persistent or worsening postprandial vomiting, dysphagia, and abdominal pain usually occur within 90 days of surgery [87]. Anastomotic strictures can be treated by various forms of endoscopic therapy, with endoscopic dilation using a balloon or bougie being the preferred treatment [71]. Studies have shown that 17% to 67% of cases responded to the first dilation, while 3% to 8% of cases required three or more dilations [33,87]. However, the presence of an ulcer at the stricture site might predispose the area for perforation during dilation. The placement of lumen-apposing metal stents for GI strictures represents another valid endoscopic approach that yields a high degree of technical and early clinical success [88]. However, when endoscopic techniques fail to treat anastomotic strictures, surgery may be required. In such cases, revisions are often performed laparoscopically, although the procedure can be technically challenging [33]. Rarely, a bypass reversal is necessary.

## 7. Surveillance

Surveillance of MU should be employed to document successful healing or to diagnose recalcitrant ulcers. Upper endoscopy should be performed 8–12 weeks after treatment initiation to evaluate the ulcer bed. Ulcers can often be completely healed and eradicated, even following the repair of a perforated ulcer alone [8].

## 8. Recurrence

Management of recurrent MU following previous repair, again begins with risk factor modification, including nicotine cessation, *H. pylori* eradication, and cessation of NSAID use. Patients may also be evaluated for other causes of ulceration, such as the presence of Zollinger–Ellison syndrome. The literature presents varying recurrence rates of MU after surgical management [15,22]. For instance, in Chang’s study, the recurrence rate was 0% after the first year, whereas Di Palma’s series reported a recurrence rate of 57% [10,69].

There are currently no established guidelines for the surgical management of recurrent MU. However, some approaches have been suggested, such as the re-revision of the GJ, the reversal of the bypass to normal anatomy, or the resection of the pouch and esophagojejunostomy [10]. Bacoeur–Ouzillou et al. found that 8 out of 15 surgeries required a revision of the GJ, with 3 patients experiencing recurrence and requiring further surgery. Total gastrectomy may be recommended to eliminate acid production as well as the main risk factors for recurrence, such as tobacco and NSAID use [57]. Endoscopic coverage of the ulcer bed by either endoscopic suturing or stent deployment may be a feasible alternative for high-risk patients, those who have experienced recurrence following previous revision, or for select patients who would otherwise require an esophagojejunostomy [74].

## 9. Conclusions

In conclusion, MU after RYGB is a potential complication that can occur due to multiple factors. The pathophysiology of MU is multifactorial, and its diagnosis relies on a high index of suspicion based on the clinical presentation followed by radiologic or endoscopic findings. A thorough diagnostic workup is essential to confirm the diagnosis, rule out other potential causes, and determine the severity of the ulcer. Management strategies for MU include lifestyle modifications, proton pump inhibitors +/− sucralfate, endoscopic suturing and/or stenting, and revisional surgery, in the most severe cases. Close follow-up and early intervention are crucial to minimizing the risk of complications and improving patient outcomes. Further research is needed to better understand the mechanisms underlying MU.

## Data Availability

Not applicable.
